# An Analysis of Laryngomalacia and Its Interplay With Obesity and Obstructive Sleep Apnea in Pediatric Inpatients

**DOI:** 10.7759/cureus.45313

**Published:** 2023-09-15

**Authors:** Dean G Kennedy, Nicholas R Wilson, Amos Mwaura, Jonathan M Carnino, Jessica Levi

**Affiliations:** 1 Otolaryngology – Head and Neck Surgery, Boston University School of Medicine, Boston, USA; 2 Otolaryngology – Head and Neck Surgery, Boston Medical Center, Boston, USA

**Keywords:** risk stratification, pediatric airway, obstructive sleep apnea, obesity, laryngomalacia

## Abstract

Objective

This study aimed to investigate the potential relationship between laryngomalacia and obesity as well as explore the interplay between laryngomalacia and obstructive sleep apnea using the Kids' Inpatient Database (KID) for the year 2016.

Methods

The Healthcare Cost and Utilization Project (HCUP) KID for 2016 provided a dataset for analysis. Patient demographics, diagnoses, and hospital characteristics were considered. Patients less than three years old were included due to the high prevalence of laryngomalacia in this age group.

Results

Among 4,512,196 neonatal admissions, 1,341 obesity cases and 11,642 laryngomalacia cases were analyzed. The frequency of laryngomalacia in patients with obesity was 578.1% higher than in the general population. Patients with obstructive sleep apnea (OSA) exhibited a 5,243.2% increase in laryngomalacia frequency compared to the overall population. Combining obesity and laryngomalacia resulted in a 6,738.5% increase in OSA frequency.

Conclusions

This study identified a significant correlation between obesity and increased laryngomalacia risk. The findings have important clinical implications for pediatric care, emphasizing the need to prevent childhood obesity to reduce laryngomalacia risk. Additionally, understanding these risk factors enables better risk stratification for laryngomalacia and potential OSA development.

## Introduction

Laryngomalacia is a congenital abnormality characterized by the inward collapse of supraglottic structures during inspiration, leading to airway obstruction. This condition, which is the leading cause of stridor in infants, affects 40% to 75% of neonates and causes life-threatening complications in 20% of these cases [1,2]. Patients affected by laryngomalacia often experience challenges with breathing while feeding due to the obstruction in the air passage [3], and this increased metabolic demand necessary to coordinate these actions can be so severe that it leads to weight loss and failure to thrive [4]. In nearly 20% of infant laryngomalacia cases, patients exhibit life-threatening symptoms that require surgical intervention [1]. Given the significant implications, early identification of laryngomalacia is critical to ensuring timely intervention and management.

The prevalence of obesity (as defined as BMI ≥ 95th percentile for age and sex) in children has consistently risen worldwide over the past five decades [5,6], affecting not only low-income countries but also middle-income and high-income countries as well [5]. Childhood obesity has long been linked to many cardiometabolic and psychological issues [7-9]. Despite many studies investigating comorbidities related to childhood obesity, its correlation with laryngomalacia remains unexplored. It’s worth noting that both laryngomalacia and obesity are reported contributors to obstructive sleep apnea in children [10,11], but no relationship between the two has been established to date.

This study aims to investigate the potential relationship between laryngomalacia and obesity as well as the co-relationship between laryngomalacia and obstructive sleep apnea using the Kid Inpatient Database (KID) for the year 2016. By examining this association, we hope to contribute to the early identification and management of laryngomalacia and shed light on the potential interplay between laryngomalacia, obesity, and obstructive sleep apnea.

## Materials and methods

Database selection

The database used for analysis was the Healthcare Cost and Utilization Project (HCUP) KID for the year 2016. This database is part of a collection offered by HCUP every three years, with the latest dataset at the time of publication being the year 2019 [12]. The database is sponsored by the Agency for Healthcare Research and Quality [12]. The KID database includes a large sample of pediatric inpatient admissions and discharges across the United States, which allows for the analysis of rare conditions and congenital anomalies such as laryngomalacia. Data recorded in the database includes primary and secondary diagnoses, procedure codes, patient demographics (gender, race, payer status), hospital characteristics (regional, rural, or urban status), in-hospital births and mortality, length of stay, and charges. The SPSS Statistics version 27 (IBM Corp., Armonk, NY, USA) was used for all data analysis.

Patient selection

The KID database encompassing the 2016 calendar year was the first of the series to use the International Statistical Classification of Diseases and Related Health Problems, 10th Revision (ICD-10) coding [13]. Based on the ICD-10 coding, we created binary variables for the presence or absence of a diagnosis of laryngomalacia, overweight and obesity, and obstructive sleep apnea. In this database, the age range for the admissions ranged from zero to 20, but for this study, only children less than three years of age were included, as this constituted 86.3% of admissions containing an associated diagnosis of laryngomalacia.

Data analysis

All data was weighted using the validated HCUP database weighting method to normalize the data to the population in the United States, as KID guidelines report only a portion of all pediatric hospital admissions. Categorical variables were analyzed using Pearson’s chi-square test to assess for differences in laryngomalacia cases by season and hospital region. A p-value less than 0.05 was the statistical cut-off for significance for the analysis in this study.

## Results

General results

In total, there were 1,844,531 inpatient pediatric patients, consisting of children less than three years old. A validated HCUP weighting system was used to transform these admissions to estimate national neonatal admission data to be 4,512,196 inpatient pediatrics visits for children aged two years old or less. A total of 1341 obesity admissions were obtained from the database to form the group for analysis, with a frequency of 29.7 per 100,000. As for laryngomalacia admissions, 11,642 were obtained from the database to form the group for analysis, and the frequency was found to be 258.0 per 100,000. Around 10,043 obstructive sleep apnea admissions were obtained from the database to form the group for analysis, and the frequency was found to be 222.6 per 100,000.

Obesity and laryngomalacia

The frequency of laryngomalacia admissions per 100,000 for patients with and without obesity (as an ICD-10-designated diagnosis) is shown in Table 1.

**Table 1:** Frequency of laryngomalacia in patients with and without obesity

With obesity	Laryngomalacia per 100,000	Laryngomalacia admissions	Total admissions
Yes	1491.4	20	1341
No	257.6	11,622	4,510,855

The frequency of laryngomalacia admissions in patients with obesity was significantly increased (p < 0.005). The frequency of laryngomalacia admissions in patients with obesity was found to be 578.1% greater than the general population. The frequency of laryngomalacia admissions in patients without obesity was found not to have a significant difference from the overall population.

Laryngomalacia and obstructive sleep apnea

The frequency of laryngomalacia admissions per 100,000 for patients with and without obstructive sleep apnea is shown in Table 2.

**Table 2:** Frequency of laryngomalacia in patients with and without obstructive sleep apnea

Obstructive sleep apnea	Laryngomalacia per 100,000	Laryngomalacia admissions	Total admissions
Yes	13,527.4	1575	11,643
No	188.2	8469	4,500,554

The frequency of laryngomalacia admissions in patients with obstructive sleep apnea was significantly increased (p < 0.005). The frequency of patients without laryngomalacia in patients with obstructive sleep apnea was significantly decreased (p < 0.005). The frequency of laryngomalacia admissions in patients without obstructive sleep apnea significantly decreased (p < 0.005). The frequency of laryngomalacia admissions in patients with obstructive sleep apnea was 5243.2% greater than the overall population. The frequency of laryngomalacia in patients without obstructive sleep apnea was 27.1% lower than in the general population.

Obesity and laryngomalacia as factors affecting obstructive sleep apnea

To analyze the effect of both obesity and laryngomalacia as variables that correlate with obstructive sleep apnea, the frequencies of both obesity and laryngomalacia were analyzed separately and together in relation to obstructive sleep apnea (Table 3).

**Table 3:** Frequency of obstructive sleep apnea admissions in patients with and without obesity and laryngomalacia OSA: Obstructive sleep apnea

Factors	OSA per 100,000	OSA admissions	Total admissions
- obesity, - laryngomalacia	184.6	8,306	4,499,234
+ obesity, - laryngomalacia	12,339.1	163	1321
- obesity, + laryngomalacia	13,524.9	1572	11,623
+ obesity, + laryngomalacia	15,000	3	20

The frequency of obstructive sleep apnea admissions in patients without obesity or laryngomalacia was found to be significantly decreased (p < 0.005) and was 82.9% of that of the overall population. The frequencies of obstructive sleep apnea in patients with obesity alone, laryngomalacia alone, and both obesity and laryngomalacia were all found to be increased (p < 0.005 for all). The frequencies of obstructive sleep apnea in patients with obesity alone, laryngomalacia alone, and both obesity and laryngomalacia were found to be 5543.2%, 6075.9%, and 6738.5% of the overall population, respectively.

## Discussion

This study aims to evaluate the relationship between obesity and laryngomalacia. The results show that there was a 578.1% increase in the frequency of laryngomalacia in patients with obesity compared to patients without obesity. Previous research has tied obesity to a number of airway conditions, such as obstructive sleep apnea [14,15]. The prevalence of obesity in infants under age two is estimated by the CDC to be around 9.5% [16]. This is significantly lower than the general population, which is estimated to be around 42.4% [17]. Studying a disease in a small subset of a tiny portion of the United States population is difficult to do with any statistical power; however, it has been made possible with large, national databases such as the KID.

The mechanism behind the correlation between laryngomalacia and obesity is currently unclear. Studies exploring the connection between obesity and obstructive sleep apnea have noted that the upper airways of obese individuals have a greater potential for collapse, caused in part by a great concentration of adipose tissue around the head and neck area and a reduced amount of traction on the airways when lying down [18]. Increases in the rate of laryngomalacia may be caused by similar mechanisms.

Alternatively, obesity has been linked to a plurality of other diseases, such as nutritional deficiencies and low socioeconomic status [19,20], which could serve as possible confounding variables in the correlational relationship between obesity and laryngomalacia. These potential mechanisms, as well as others, should be studied in an attempt to better understand why these two disease processes correlate.

Although the findings of this study are limited, they could still have significant clinical implications. As previously discussed, laryngomalacia is one of the most common causes of neonatal stridor, with almost 20% of laryngomalacia cases requiring urgent intervention, according to some reports [1]. Identifying risk factors for the disease can help risk stratify patients for the probability of developing laryngomalacia and any adverse events stemming from it. Future studies of laryngomalacia and its relationship to obesity can also help elucidate a mechanism to develop improved treatment modalities.

The limitations of our study include those inherent to any study of a large database. The risk of incorrect data entry cannot be excluded. Furthermore, the KID database also only captured admissions from the year 2016, and it is possible that the relationship between laryngomalacia and obesity may have changed in today’s population. An additional limitation is the lack of objective diagnostic criteria for obstructive sleep apnea and laryngomalacia listed in the KID; this leaves the possibility of misdiagnosis or underdiagnosis. Additional avenues for future research include conducting research with databases that include these criteria. Further limitations of the KID include a lack of certain data points that would be relevant to our study, including patient weight as a numerical value. For our study, we used an obesity ICD-10 code to identify patients with obesity. Having a patient's weight and height listed to calculate their BMI could be a topic of future study since it would allow for more granular analysis. Lastly, the KID contains pediatric admissions, so some cases of laryngomalacia were not captured in our analysis.

## Conclusions

The present study found an increase in the frequency of laryngomalacia in patients with obesity compared to patients without obesity. These findings contribute to bridging the current knowledge gap in the literature. The results of this study have important clinical implications regarding pediatric care. Although the causes of obesity are multifactorial, the increased risk of laryngomalacia serves as further motivation to provide resources to prevent childhood obesity. Furthermore, awareness of other risk factors for laryngomalacia will enable healthcare providers to better risk stratify patients. Future studies should continue to explore the mechanism by which obesity increases the risk of laryngomalacia, as through doing so, treatment modalities can be developed.
